# Free Cysteine Modulates the Conformation of Human C/EBP Homologous Protein

**DOI:** 10.1371/journal.pone.0034680

**Published:** 2012-04-04

**Authors:** Vinay K. Singh, Mona N. Rahman, Kim Munro, Vladimir N. Uversky, Steven P. Smith, Zongchao Jia

**Affiliations:** 1 Department of Biomedical and Molecular Sciences, Queen's University, Kingston, Ontario, Canada; 2 Department of Molecular Medicine, University of South Florida, Tampa, Florida, United States of America; 3 Institute for Biological Instrumentation, Russian Academy of Sciences, Pushchino, Moscow Region, Russia; Semmelweis University, Hungary

## Abstract

The C/EBP Homologous Protein (CHOP) is a nuclear protein that is integral to the unfolded protein response culminating from endoplasmic reticulum stress. Previously, CHOP was shown to comprise extensive disordered regions and to self-associate in solution. In the current study, the intrinsically disordered nature of this protein was characterized further by comprehensive *in silico* analyses. Using circular dichroism, differential scanning calorimetry and nuclear magnetic resonance, we investigated the global conformation and secondary structure of CHOP and demonstrated, for the first time, that conformational changes in this protein can be induced by the free amino acid l-cysteine. Addition of l-cysteine caused a significant dose-dependent decrease in the protein helicity – dropping from 69.1% to 23.8% in the presence of 1 mM of l-cysteine – and a sequential transition to a more disordered state, unlike that caused by thermal denaturation. Furthermore, the presence of small amounts of free amino acid (80 µM, an 8∶1 cysteine∶CHOP ratio) during CHOP thermal denaturation altered the molecular mechanism of its melting process, leading to a complex, multi-step transition. On the other hand, high levels (4 mM) of free l-cysteine seemed to cause a complete loss of rigid cooperatively melting structure. These results suggested a potential regulatory function of l-cysteine which may lead to changes in global conformation of CHOP in response to the cellular redox state and/or endoplasmic reticulum stress.

## Introduction

C/EBP Homologous Protein (CHOP) is a nuclear protein that acts as both a dominant-negative inhibitor of CCATT/enhancer binding proteins (C/EBPs) and a transcriptional activator of Activating Protein–1 (AP-1) [1]. This 29 kDa protein contains two functional domains, an N-terminal transcriptional activation domain and a C-terminal basic-leucine zipper (bZIP) domain [1]–[3]. Two adjacent serine residues (Ser79 and Ser82) can be phosphorylated by the p38 MAP kinase family [4]. CHOP plays an integral role in one of the branches of the unfolded protein response (UPR) which is initiated in response to endoplasmic reticulum (ER) stress [5]–[8], and also occurs in response to factors such as nutrient starvation, calcium depletion, inflammation, glucose deprivation, and viral infection [5], [9]–[11]. This response promotes cellular recovery, and leads to apoptosis if recovery is not possible. While the exact signaling mechanism underlying ER stress-induced apoptosis is poorly understood, CHOP has been implicated in two potential mechanisms. As a transcriptional regulator, CHOP plays a role in linking ER stress to alterations in gene expression, and it is a major pro-apoptotic component of the UPR [1], [12], [13]; it represses Bcl2 and induces apoptotic proteins such as Gadd34, Dr5, Bim and Trb3 [3], [14]–[17]. CHOP has also been shown to induce ER oxidase 1α (ERO1α) which, in turn, activates IP3R1, the ER calcium-release channel found to be crucial for mediating events triggered by cytoplasmic calcium. The CHOP-ERO1α pathway directly promotes hyperoxidizing conditions in the ER lumen and results in cytoplasmic oxidative stress by inducing NADPH oxidase subunit Nox2 as well as by generation of reactive oxygen species (reviewed in [8]). Deletion mutant analysis of CHOP revealed that bZIP domain is important for CHOP-induced apoptosis [18], [19].

Despite its involvement in the UPR, to depict CHOP as purely a pro-apoptotic transcription factor is an oversimplification. Rather, CHOP has been suggested to mediate cell- and context-specific responses to stress that are either adaptive or maladaptive [20]. CHOP expression is induced in myoblasts to prevent premature differentiation by binding to the upstream regulatory region of the *myod* gene and repressing its transcription. Its activity reduces histone acetylation at the MyoD enhancer region through an interaction with histone deacetylase I (HDAC) [21]. In oligodendrocytes, CHOP appears to protect against death while, in contrast, CHOP promotes demyelination in Schwann cells without cell death [20].

CHOP induction has been implicated in diseases such as homo-cysteinemia, brain ischemia, human sarcomas, and neurodegenerative disorders [3], [22]–[25]. It may contribute to heart attacks by promoting apoptosis of the macrophages in atherosclerotic plaques, which leads to plaque rupture [26], and CHOP-mediated apoptosis subsequently contributes to reperfusion injury [27]. CHOP also supports β-cell apoptosis in diabetes; the UPR is triggered by misfolded (pro)insulin, present due to either over-synthesis or mutations that inhibit processing, leading to CHOP-mediated apoptosis [11], [28]. Pathological effects might also be caused by CHOP's association with the immune response, through its activation of the pro-inflammatory transcription factor NF-κB [29]. Since CHOP itself is also triggered by inflammatory cytokines [30], this represents a possibly dangerous positive feedback loop when activated.

It is generally accepted that the biological function of a protein is intimately related to its three-dimensional structure. Indeed, a common approach to discerning the potential function of an unannotated protein is by comparison with the unique structural folds of known proteins. However, within the last decade, it has become increasingly clear that a significant fraction of the universal proteome consists of intrinsically disordered proteins (IDPs) or proteins with intrinsically disordered regions (IDRs) [31]–[35]. IDPs exist as dynamic ensembles in which the atom positions and backbone Ramachandran angles vary significantly over time with no specific equilibrium values, and typically undergo non-cooperative conformational changes [31], [35]–[46]. Numerous studies have demonstrated that many proteins and regions thereof function in this intrinsically disordered (ID) state [35]–[37], [47]–[50], challenging the classical notion that the function of a protein is predefined by its three-dimensional structure. Proteins with ordered structures have mainly evolved for efficient catalysis [51], whereas IDPs are typically involved in regulation, signaling and control pathways [52]–[54] as the flexibility of disordered regions allow interactions with several partners, promoting more efficient use of the same protein in multiple pathways [55]. It is, thus, not surprising that IDPs have been implicated in various diseases such as cancer, cardiovascular disease, amyloidosis, and neurodegenerative diseases [47], [56].

Previously, we reported that CHOP is an IDP and that its disordered N-terminal domain mediates oligomerization, which plays a key role in its function in both inhibition of Wnt/Tcf signaling as well as stimulation of c-Jun and sucrase-isomaltase reporter activity in intestinal colon cancer cells [57]. IDPs such as CHOP can bind partners with both high specificity and low affinity [58], thus fulfilling the two fundamental requirements of signaling interactions – specificity and reversibility [59]. Furthermore, IDRs possess tremendous binding diversity, able to interact specifically with numerous partners, including proteins, nucleic acids, polysaccharides and small molecules [35]–[37], [40], [41]. This makes them ideal for playing important roles in the exquisitely complex network of protein-protein interactions, as they can achieve very high connectivity levels. As such, proteins such as CHOP are known as “hub" proteins and it is precisely this intrinsic disorder which is suggested to play a crucial role in hub protein functions [53], [54].

The binding promiscuity of IDPs, which gives them their important role in signaling and regulatory functions, also demands that they be under strict regulation and carefully monitored [55], [56], [60]. Moreover, understanding this regulation is important as irregular expression of IDPs is associated with several disease states [47], [55], [60]. As such, the availability of IDPs is strictly regulated at various levels from transcript synthesis to protein degradation. Multiple mechanisms control availability at the level of transcription and translation, including higher miRNA target sites and higher mRNA decay rates, greater ubiquitination sites, and increased susceptibility for degradation, all of which result in many IDPs being present at relatively low levels and for shorter periods of time than their structured counterparts. While this may be the case for the majority of IDPs, some may be required at high levels or for prolonged periods of time in response to certain conditions or at certain phases of the cell cycle, or even required at high levels during the entire cell's lifetime. Some IDPs can be stabilized by association with “nanny proteins" which bind to IDRs and facilitate degradation, while others are stabilized by becoming part of stable complexes or by interaction with other partners. Post-translational modifications, such as phosphorylation, can influence the balance between the bound and unbound states of these proteins (reviewed in [55]). On the other hand, another concern originates from the flexibility and promiscuity of IDPs, which leads to the question of what mechanisms are in place to avoid unwanted interactions. One model set forth is the functional misfolding of “sticky" preformed secondary structure elements such that they are sequestered and excluded from the environment as a means of “hiding" them from unwanted binding interactions (reviewed in [56]).

In this study, we continue our structural characterization of CHOP by investigating the regulation of this protein via analysis of the effects of various thiols on its conformation. Levels of CHOP have been shown to increase with those of homocysteine and cysteine, conditions indicative of homocysteine stress [61]. Furthermore, CHOP translocation occurs under redox stress [62]. Translocation through membranes is thought to be optimal when a protein is in a “molten globule" state, i.e. a state combining structural features of ordered and disordered conformations [63]. Since CHOP is a prominent resident of the ER and an aberrant-ER marker, and since intracellular thiols such as cysteine, homocysteine, and glutathione play critical roles in the regulation of ER protein synthesis and folding [64], in this study we examined whether thiols could affect CHOP structure and potentially play a regulatory role. We demonstrate that l-cysteine, but not homocysteine or glutathione, affects the global conformation of CHOP in a concentration-dependent manner. Circular dichroism (CD), differential scanning calorimetry (DSC), and nuclear magnetic resonance (NMR) studies were employed to show that conformational changes in CHOP can be induced by the free amino acid l-cysteine (but not d-cysteine). This finding is discussed as a plausible signal transduction mechanism during ER stress-mediated apoptosis.

## Results

### Disorder analysis of CHOP

**Figure 1:**
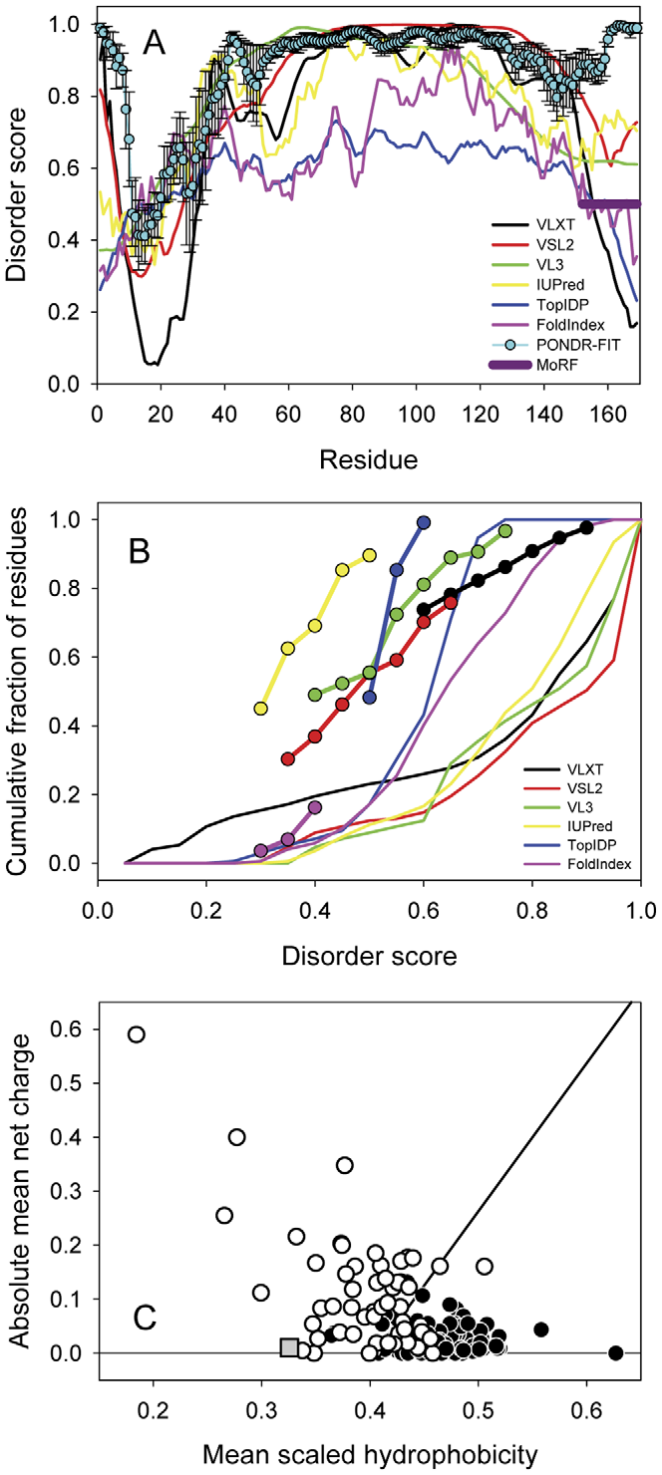
*In silico* disorder analysis of CHOP using various prediction programs (IVLXT, VSL2, VL3, IUPred, TopIDP, Foldindex and PONDR-FIT). (A) Distribution of predicted disorderedness per-residue. (B) Cumulative distribution function (CDF) analysis of CHOP. (C) Charge-hydropathy analysis of CHOP (grey) in comparison to natively folded proteins (closed circles) and natively unfolded proteins (open circles).

Previously, we reported that CHOP is an IDP based on *in silico* amino acid sequence analyses, NMR and CD spectroscopy [57]. For the present study, a more comprehensive analysis was performed to characterize the “disorderedness" of the protein. There is vast structural variability of IDPs, ranging from being completely unstructured, to containing some elements of secondary and/or tertiary structure, to molten globular proteins with well-developed secondary structure and high compactness degree, to domains (both ordered and disordered) containing highly flexible linkers [45], [65]–[67]. The various computational tools available tend to use different criteria, attributes, algorithms and databases to predict disorder and, up to now, there is no single program which is accurate enough to be completely trusted for reliability [68]–[71]. Consequently, we employed several different programs (i.e., PONDR VLXT [72], [73], PONDR VSL2 [74], PONDR VL3 [75], IUPred [76], TopIDP [77], Foldindex [78] and a meta predictor PONDR-FIT [79]) for the *in silico* analysis of the amino acid sequence (Figure 1A), which consistently predicted that CHOP is expected to be highly disordered over most of its length when looking at the disorder predicted on a per-residue basis. The C-terminus of CHOP is identified as a helical molecular recognition feature (MoRF), a disordered interaction motif that has the tendency to adopt an α-helical conformation on binding of a target protein [80].

Given the extreme conformational variability of IDPs, ranging from collapsed molten globule-like disorder to extended coil-like disorder, and given the coexistence of ordered and disordered proteins, a binary classification can be performed to categorize the protein as entirely disordered or ordered. One methodology commonly used is a cumulative distribution function (CDF) analysis, which improves the accuracy of disorder prediction on the level of the whole protein [65]. In CDF analysis, a protein is predicted to be mostly disordered if the majority of the resultant curve is located below the boundary between ordered and disordered proteins. With all of the algorithms utilized for this analysis, the majority of the respective CDF curves for CHOP resided below the boundary (Figure 1B), thus implying that CHOP is expected to be disordered as a whole. Finally, an additional binary classification was performed using a charge-hydropathy plot to determine the linear boundary which separates compact and extended proteins (Figure 1C) [81]. In the charge-hydropathy plot, CHOP clearly falls in the region corresponding to the natively unfolded proteins (i.e. IDPs with extended disorder), thus further supporting its definition as a disordered protein.

### CD analysis of CHOP secondary structure

Since CHOP is considered to serve as a potential “hub" protein, we attempted to investigate means by which it may be regulated. We had already established that the disordered N-terminal domain of CHOP mediates its oligomerization, its putative native state, which plays a key functional role in inhibition of Wnt/Tcf signaling as well as stimulation of c-Jun and sucrase-isomaltase reporter activity in intestinal colon cancer cells [57]. Given that (1) increases in levels of both homocysteine and cysteine have been associated with increases in CHOP levels [61], (2) that its translocation occurs under redox stress [62] and that, generally, translocation through membranes is presumably optimal when a protein is in a “molten globule" state (i.e. a state characterized by a combination of features typical for ordered and unfolded proteins) [63], (3) that CHOP is a prominent resident of the ER and an aberrant-ER marker, and (4) that intracellular thiols such as cysteine, homocysteine and glutathione play critical roles in the regulation of ER protein synthesis and folding [64], we investigated the effect of various thiols on CHOP structure. Initially, circular dichroism (CD) was used to investigate changes in secondary structure. Titration of CHOP with homocysteine, glutathione and d-cysteine showed no change in conformation as measured by CD (data not shown). However, l-cysteine affected conformation.

**Figure 2:**
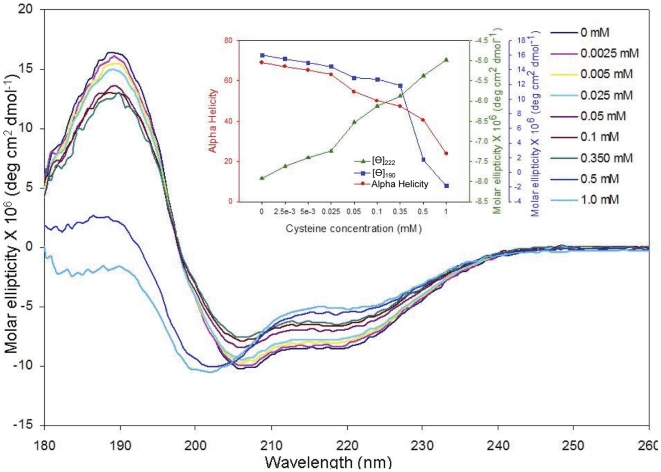
Global conformational changes in CHOP in the presence of l-cysteine measured using CD analysis. Far -UV CD spectra of CHOP were recorded on an OLIS RMS CD spectrophotometer at 25°C. The sample contained 0.16 mg/mL CHOP in 10 mM phosphate buffer, pH 7.0, and was titrated with varying concentrations of l-cysteine, as described in the figure legend. For each, a total of 8 scans were collected, each corrected against buffer and l-cysteine blanks, and averaged. Inset: Graph depicting the decrease in [θ]_190_, [θ]_222_, and secondary structure (alpha helicity) content in the presence of varying concentrations of l-cysteine.

Far-UV analysis on native CHOP revealed an intriguingly high percentage of α-helical content for an apparently disordered protein (Table 1). Our previous studies strongly predicted that that C-terminal domain of CHOP contains a long coiled-coil region [57]. Furthermore, it has been determined that the native protein exists in an oligomeric state, formation of which is mediated by its N-terminal disordered region, suggesting that CHOP oligomerization may involve the formation of an α-helical coiled-coil. Titration of the native protein with l-cysteine resulted in changes of the shape and intensity of the CHOP far-UV CD spectrum (Figure 2). Notably, increasing amounts of l-cysteine resulted in changes in the characteristic bands at 222, 208 and 190 nm, with the appearance of a new band at 200 nm, implying a decrease in ordered secondary structure content. More specifically, changes at 222 and 190 nm were attributed to a decrease in α-helical content (Figure 2, inset). This hypothesis was supported by the deconvolution of the CD spectra [82], which revealed a concomitant decrease in α-helical content with increasing concentrations of l-cysteine, dropping from 69.1% to 23.8% in the presence of 1 mM of l-cysteine (Table 1). An increase in coil content was observed as well, implying that addition of l-cysteine resulted in a transition of the CHOP protein to a more disordered state.

**Table 1:** CD-based secondary structure measurements of CHOP.^a^

l-Cysteine (mM)	Helix (%)	Antiparallel (%)	Parallel (%)	Turn (%)	Coil (%)
0	69.1	3.0	2.5	12.3	10.2
0.0025	67.1	3.2	2.7	12.6	10.8
0.005	65.3	3.4	2.9	12.8	11.5
0.025	63.1	3.6	3.1	13.1	12.3
0.05	54.5	4.7	4.1	14.2	16.1
0.1	50.1	5.4	4.8	14.8	18.3
0.35	47.3	5.9	5.2	15.2	19.9
0.5	40.5	6.6	7.2	15.8	28.2
1	23.8	12.9	5.4	24.8	32.8

a Measurements of CHOP were performed in 10 mM phosphate buffer (pH 7.0, 25°C), in the presence of increasing concentrations of l-cysteine. Calculations were performed using CDNN [108].

**Figure 3:**
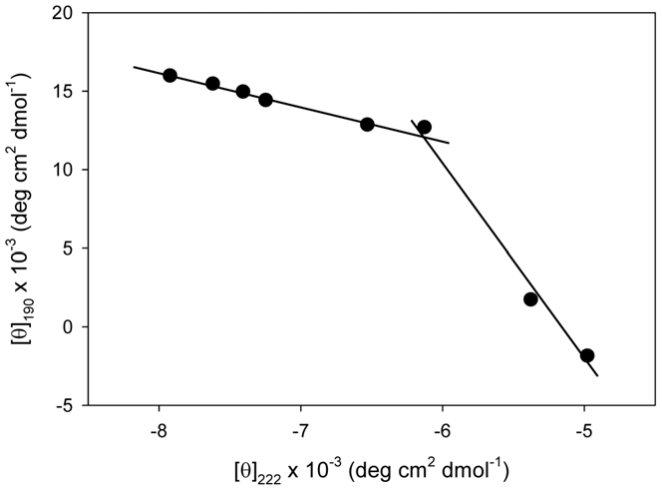
Phase diagram showing the dependence of [θ]_190_ vs. [θ]_222_ on increasing l-cysteine concentrations. Data was adapted from Figure 2. Note the two-state linear regression, which is representative of consecutive conformational transitions in CHOP.

To analyze further the mechanism by which l-cysteine induced unfolding, a phase diagram was constructed from the CD data [83]. This transformation is a highly sensitive technique for detecting intermediate conformational states [83]–[86]. Such a phase diagram examines the relationship between the spectral intensities of two wavelengths under different experimental conditions while a protein is undergoing structural transformations (see Methods and Materials) [83]. Given the changes observed in both θ_190_ and θ_222_ with increasing amounts of l-cysteine, we analyzed the relationship between these two parameters (Figure 3). The relationship between two spectral parameters is expected to be linear if changes in the protein's environment results in an all-or-none transition between two conformations or, in our case, a “melting" of the structure. In contrast, our analysis revealed the existence of two linear dependencies, which is reflective of sequential conformational transitions in CHOP.

**Figure 4:**
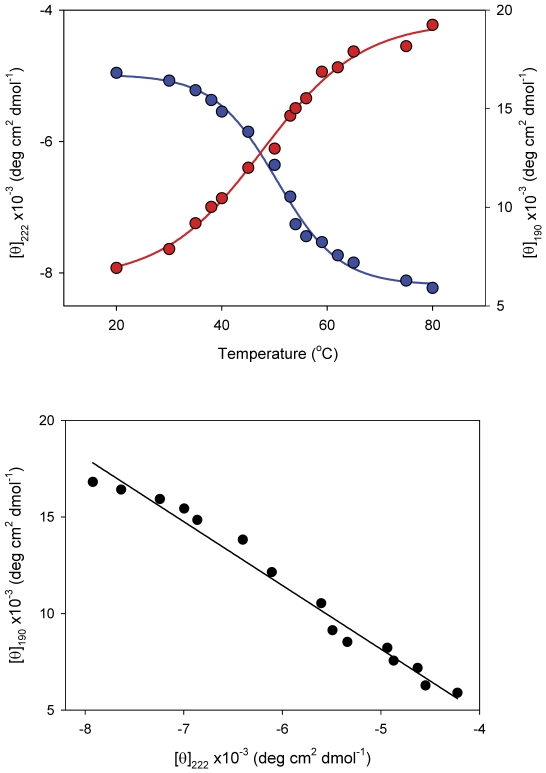
Thermal denaturation of CHOP measured using far-UV CD. Top panel shows a graph depicting the changes in [θ]_190_ and [θ]_222_ with at increasing temperatures. Far-UV CD spectra of CHOP were recorded at increasing temperatures (20°C–80°C) on an OLIS RMS CD spectrophotometer with a Pt100 thermal probe fitted right under the cuvette. Temperatures in the cuvette were controlled using a Julabo F30-C circulating water bath. A total of 8 scans were conducted at each temperature and averaged, with 10 minutes of equilibration time between each experiment. Bottom panel depicts a phase diagram showing the dependence of [θ]_190_ vs. [θ]_222_ on temperature. Note that the linear regression is representative of an all-or-none transition.

In order to confirm that the l-cysteine-induced conformational changes in CHOP differ from those involved in temperature-induced melting of this protein, the far-UV CD spectra were taken at increasing temperatures, ranging from 20°C to 80°C. This analysis revealed a gradual loss of intensity with increasing temperature reflective of protein denaturation. Specifically, increasing temperature resulted in concomitant changes in the intensities at 190 and 222 nm, reflecting a loss of α-helicity, as depicted by the characteristic sigmoidal curves describing the temperature dependencies of these molar ellipticities (Figure 4, top panel). Notably, the observed changes occurred over a relatively large temperature range (∼30°C–75°C). Transformation of this data into a phase diagram (Figure 4, bottom panel) demonstrated a linear relationship between θ_190_ and θ_222_, implying that, in contrast to l-cysteine-induced conformational changes, temperature-induced denaturation of CHOP can be described as an all-or-none transition. Thus, the loss of structure caused by the addition of l-cysteine is likely not due to a “melting" of the protein as seen in temperature-denaturation, but results in specific conformational changes leading to its loss of secondary structure.

### Effect of l-cysteine on thermal denaturation of CHOP

**Figure 5:**
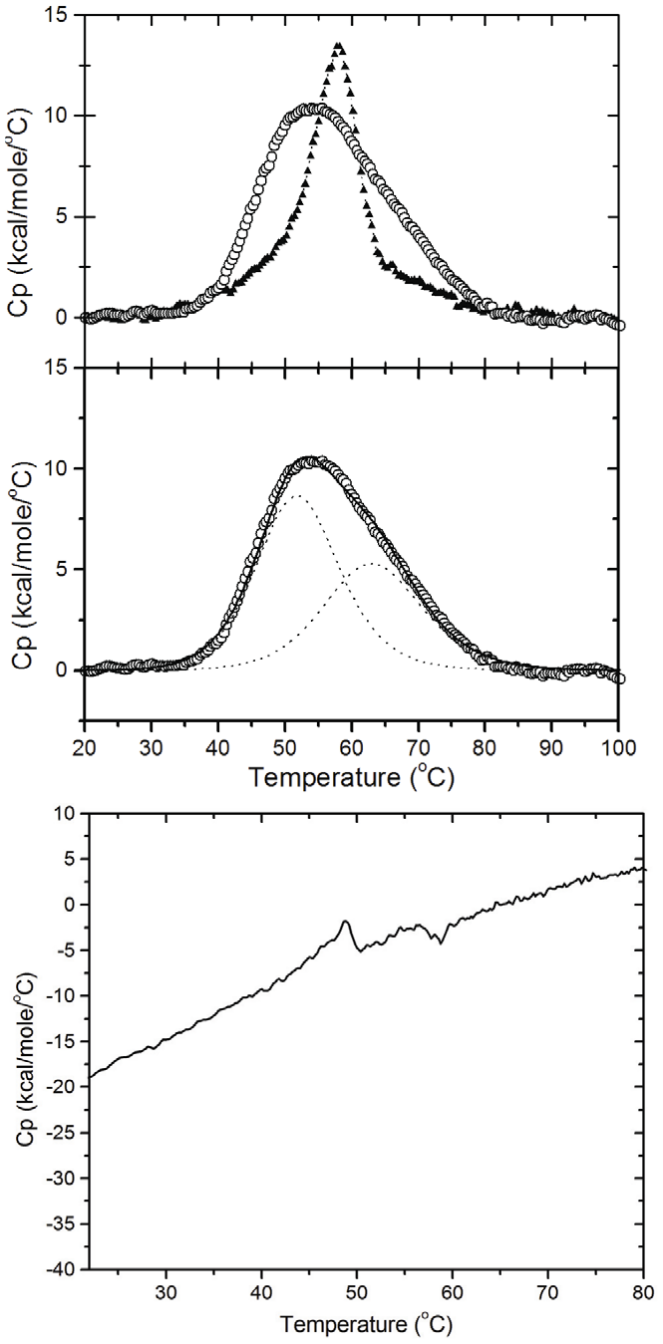
Thermal denaturation profile of CHOP measured using DSC. Top panel: Normalized DSC scan of CHOP in 5 mM phosphate buffer (pH 7.0) in the absence (solid triangle) or presence (open circle) of 80 µM l-cysteine. Middle panel: Two-state fitting (dashed line) of normalized DSC scan of CHOP in presence of 80 µM l-cysteine. Bottom panel: Normalized DSC scan of CHOP in presence of 4 mM l-cysteine. The heating rate was 45°C per hour. Approximately 10 µM CHOP was used for each run.

Further analysis of the effect of l-cysteine on conformational change was performed by studying the thermal denaturation of CHOP in the presence and absence of l-cysteine by differential scanning calorimetry (DSC). The variation in heat capacity associated with protein unfolding is primarily due to the changes in hydration of side chains buried in the native state, which become exposed to the solvent in the denatured state [87]. In the absence of l-cysteine, thermal denaturation of CHOP resulted in a sharp peak of heat absorption (Figure 5, top panel), with a melting temperature of 57°C. Interestingly, in the presence of 80 µM l-cysteine (i.e. a cysteine∶CHOP ratio of 8∶1), the CHOP heat absorption peak widened and became asymmetrical, suggesting that, under these conditions, thermal denaturation is a multi-step process. Deconvolution of the normalized heat absorption peak (Figure 5, middle panel) shows the presence of two overlapping peaks, suggesting the presence of at least two individual calorimetric units with melting temperatures of 52°C and 63°C, respectively. Further increase in the l-cysteine concentration to 4 mM (Figure 5, bottom panel), led to the complete disappearance of the cooperative heat absorption, suggesting that under these conditions (i.e. a cysteine∶CHOP ratio of 400∶1), CHOP has lost any rigid structure that it may have had in its native state [88].

### NMR analysis of l-cysteine-induced structural changes in CHOP

**Figure 6:**
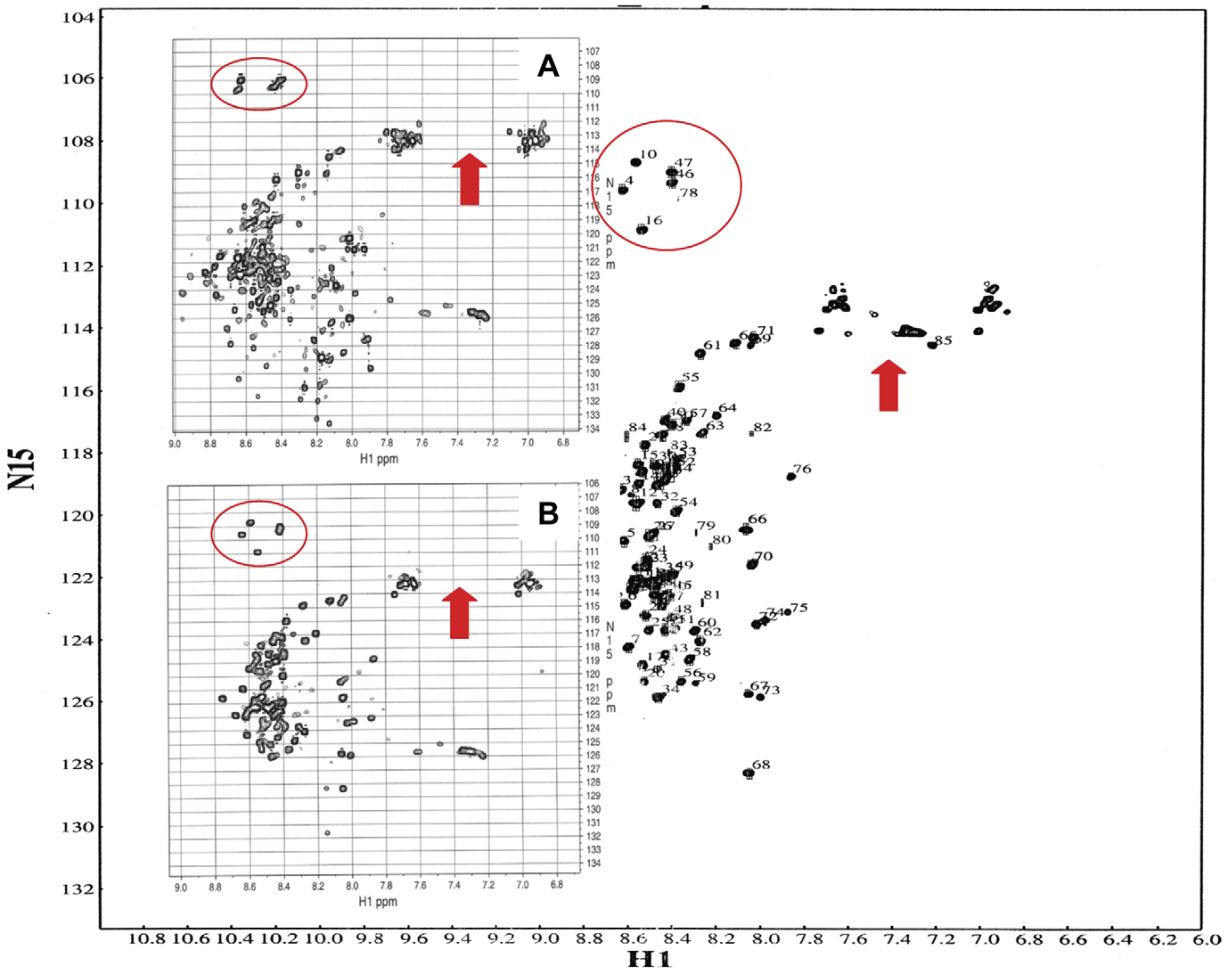
Two-dimensional ^1^H-^15^N-HSQC spectra of CHOP in the absence (background) and presence of 2 mM (inset A) and 8 mM (inset B) l-cysteine at 4°C. Circles identify backbone ^1^H-^15^N resonances affected by l-cysteine while arrows identify a region comprising side chain amide resonances affected l-cysteine.

To investigate further the structural changes resulting from the addition of l-cysteine to CHOP, we utilized NMR spectroscopy to monitor the conformation of CHOP in the absence and presence of the amino acid (Figure 6). The limited dispersion of resonances in the proton dimension of the ^1^H-^15^N HSQC spectrum of CHOP is indicative of an unstructured protein. Addition of l-cysteine resulted in chemical shift changes in resonances corresponding to glycine backbone amide groups and asparagine and glutamine side chain amide groups, further supporting a change in CHOP conformation in the presence of l-cysteine.

## Discussion

In our previous study, we reported that CHOP is an IDP that contains extensive disordered regions and self-associates in solution. The N-terminal region was found to mediate this oligomerization and was vital for the functional roles of CHOP in inhibiting Wnt/TCF signaling and stimulating c-Jun and sucrase-isomalatase reporter activity in intestinal colon cancer cells [57]. We expanded our structural analysis of CHOP, using a comprehensive *in silico* analysis to provide further support that it is an IDP. Since CHOP is a “hub" protein that has integral and multiple roles in the UPR pathway, we looked at means by which CHOP could be regulated. A unique functional feature of IDPs is their binding promiscuity since their highly dynamic and flexible structure allows a single region of disorder to adopt different conformations using the same amino acids to varying extents in order to bind a variety of partners [54], [89].

The fact that intracellular thiols play critical roles in the regulation of ER protein synthesis and folding, and that CHOP is involved in responding to ER stress further supported our strategy for screening thiol compounds. Using a combination of CD, DSC and NMR, we were able to demonstrate that addition of free l-cysteine is able to modify the conformation of CHOP to a more “disordered" state. This was not a general effect of thiols as neither d-cysteine, homocysteine nor glutathione had any effect on the protein secondary structure. CD analysis showed an α-helical content of 69% in the native state which was reduced in a concentration dependent manner until reaching 24% in the presence of 1 mM l-cysteine. Given that CHOP is an IDP, such a high α-helical content in its native state in the absence of l-cysteine seems counter-intuitive. However, we have demonstrated that CHOP exists as an oligomer in solution, and that its C-terminus is strongly predicted to form a coiled-coil, which may account for the α-helical signal observed by the CD analysis.

The addition of l-cysteine appears to cause a complex, multistage transition to a more disordered state, as demonstrated by the analysis of the CD-based phase diagrams. This is in a contrast to the thermal melting of CHOP in the absence of l-cysteine, which appears to be a two-state process, as further confirmed by the DSC analyses of thermally denatured protein in the presence and absence of l-cysteine. While the native protein experienced a sharp transition from a native to an “unfolded" state, the addition of l-cysteine appeared to cause the formation of two independent calorimetric units. Furthermore, excess l-cysteine resulted in the complete disintegration of rigid, cooperatively melting structure of the protein. While this marks a serendipitous discovery of cysteine modulating the conformation of CHOP, its ability to do so is not entirely novel. The thiol group of cysteine, usually important in the antioxidation of ROS and free radicals [90], [91], was found to act as a radical mediator, inducing conformational changes in the secondary structure of bovine serum albumin in the presence of UV-B irradiation which was suggested to cause a progressive transformation from α-helix to an intermolecular β-sheet structure [92].

The specific ability of l-cysteine, and not glutathione, to cause such effects is not unusual. While the glutathione/glutathione disulfide (GSH/GSSG) redox pair does constitute the most prominent cellular redox pair [93], it is the cysteine/cystine (Cys/CySS) couple which predominates in the plasma [94]. Furthermore, the steady-state redox potential of the two pairs is regulated independently of each other [95]–[97], with that of plasma Cys/CySS being oxidized during oxidative stress [98], [99]. Indeed, selective oxidation of Cys/CySS was observed, with no change in the oxidation state of GSH/GSSG, in the acute phase of endotoxin-induced lung injury in mice [100].

There are several accounts pointing to the physiological importance of cysteine. In the aforementioned Iyer *et al.* study reporting on endotoxin-induced lung injury in mice, enhanced oxidation of Cys, altered transport of Cys and CySS and decreased food intake each contributed to the oxidation of the plasma Cys/CySS redox state in endotoxemia [100]. In yeast, exogenous addition of homocysteine and cysteine was found to inhibit growth, not due to oxidative stress, but putatively due to ER stress resulting in the upregulation of transcription of metabolic genes as a strategy to reduce intracellular thiol concentrations [64]. We have already stated that levels of CHOP are modulated under homocysteine stress [61] while its translocation occurs under redox stress [62]. CHOP is a prominent resident of the ER and an aberrant-ER marker, and intracellular thiols such as cysteine, homocysteine and glutathione play critical roles in the regulation of ER protein synthesis and folding [64]. Furthermore, translocation through membranes is thought to be optimal when a target globular protein is converted into a “molten globule"-like conformation and possesses the necessary combination of structural features typical for folded and unfolded proteins [63]. Plasma concentrations of cysteine have been reported in the range of 214–260 µM [101], [102]. There is limited information about the exact amount of cysteine present inside the ER lumen but intracellular concentrations of thiols has been shown to be in the millimolar range [103] with relatively higher concentrations of oxidized glutathione in the ER lumen to facilitate native disulfide bond formation [104]; thus, it is not unlikely for the ER lumen to have a higher localized concentration of cysteine. Of note, studies examining the cysteine-mediated growth defect in yeast by Kumar *et al.* were performed at similar and up to five times greater cysteine concentrations than in our study [64]. Thus, one may envision a scenario in which high cysteine levels as a result of ER/oxidative stress may result in CHOP adopting a more disordered conformation and dissociate from its oligomeric state into one that is more suitable for the subsequent translocation through the membrane. This may facilitate membrane translocation to where it is most needed, resulting in apoptosis, or other CHOP-induced processes. Further analysis of this hypothesis is required.

In conclusion, we have identified the specific ability of l-cysteine, and not homocysteine or glutathione, to cause global conformational change in CHOP, resulting in an increase in its disorder. Thus, this represents a means by which IDPs, such as CHOP, can be regulated to switch between different structural states of varying disorder, likely serving different functional roles.

## Materials and Methods

### *In silico* analysis of CHOP

Disorder predictions for CHOP were performed using the IVLXT, VSL2, VL3, IUPred, TopIDP, Foldindex and PONDR-FIT programs [31], [41]–[46].

### Cloning, expression and purification of CHOP

Cloning, expression and purification of CHOP were performed as described previously [57].

### Circular dichroism (CD)

CD analyses were performed at 25°C, using an OLIS RSM CD spectrophotometer. Far-UV and near-UV CD analyses were performed using CHOP protein concentrations of 10 µM and 25 µM, and path-lengths of 0.1 cm and 1 cm, respectively. The spectrophotometer was purged with pure N_2_ during the course of the experiment. Each CD plot was calculated from an average of eight accumulated scans, which were baseline-corrected using the relevant buffer. The molar ellipticity, [θ], was calculated from the observed ellipticity θ, using the equation [θ] = θ×100/*ncl*, where *n* is the number of amino acids in the protein, *c* is the molar protein concentration and *l* is the path length of the cell in centimetres. Deconvolution of the data and secondary structure analysis was performed using CDNN software [82].

### Phase diagram analysis of conformational changes

The “phase diagram" method, a highly sensitive technique for detecting intermediate conformational states from fluorescence or spectroscopic data [83], [83]–[86], was utilized to analyze changes in CHOP conformation. Values of [θ]_190_ and [θ]_222_ in the presence of varying concentrations of l-cysteine were plotted against each other. The essence of this method is to graph *I*_λ1_ versus *I*_λ2_, where *I*_λ1_ and *I*_λ2_ are the spectral intensity values measured at wavelengths λ_1_ and λ_2_ under different experimental conditions for a protein undergoing structural transformations. As spectral intensity is an extensive parameter, it will describe any two-component system by the relationship *I(λ) = α*_1_ *I*_1_ *(λ)*+*α*_2_ *I*_2_ *(λ)*, where *I*_1_ *(λ)* and *I*_2_ *(λ)* are the intensities of the first and second components, and *α*_1_ and *α*_2_ are their relative concentrations (*α*_1_+*α*_2_ = 1). It can then be derived [80] that *I*(*λ*_1_) = *a*+*bI*(*λ*_2_),
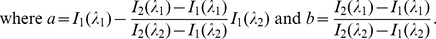
In application to protein unfolding, this predicts that 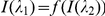 will be linear if changes in the protein's environment lead to an all-or-none transition between two different conformations. Alternatively, non-linearity of the function reflects sequential character in structural transformations. In this case, each linear portion of the 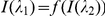 dependence will describe an individual all-or-none transition [105].

### Differential scanning calorimetry

Thermal denaturation studies were performed using a VP-DSC calorimeter (Microcal Inc., Northampton, MA) which heated the protein from 20°C to 85°C at a rate of 45°C/h. Prior to analysis, samples were dialyzed overnight in 4 L of buffer containing 10 mM Na_2_HPO_4_, 1.8 mM KH_2_PO_4_ (pH 7.3), 140 mM NaCl, and 2.7 mM KCl. The dialysis buffer was filtered using a 0.22-µm filter (Millipore, Billerica, MA) and used to obtain a baseline from several buffer-buffer scans. Protein-buffer scans were performed at a final protein concentration of 0.2 mg/mL. Protein samples were assessed for aggregation by monitoring A_340_ at various time intervals. Data analyses were performed using Microcal Origin 5.0 (Microcal Inc., Northampton, MA).

### NMR Spectroscopy

Two-dimensional ^1^H-^15^N HSQC NMR spectra of 0.4 mM uniformly ^15^N-labeled CHOP were recorded using a Varian INOVA 600 MHz spectrometer equipped with a pulse field gradient triple resonance cryoprobe at 4°C. The experiments were carried out in PBS (pH 6.8) containing 90% H_2_O/10% D_2_O, using the enhanced sensitivity pulsed-field gradient approach. Carrier frequencies of 4.77 ppm (^1^H), and 118 ppm (^15^N) were used and ^1^H chemical shifts were calibrated relative to the trimethylsilyl resonance (0.0 ppm) of 2,2-dimethyl-2-silapentane-5-sulfonate (DSS). Spectra were processed and analyzed using NMRPipe [106] and NMRview [107], respectively.
